# Effects of Physical Activity on Daily Physical Function in Chinese Middle-Aged and Older Adults: A Longitudinal Study from CHARLS

**DOI:** 10.3390/jcm11216514

**Published:** 2022-11-02

**Authors:** Yuge Tian, Zhenguo Shi

**Affiliations:** School of Physical Education, Shandong University, Jinan 250061, China

**Keywords:** physical activity, daily physical function, middle-aged and older adults, CHARLS

## Abstract

Objective: Impaired daily physical function has become a common health problem among Chinese middle-aged and elderly people. The aim of this study was to investigate the effects of physical activity on daily physical function in Chinese middle-aged and older adults. Methods: Data from 9056 participants in the China Health and Retirement Longitudinal Study (CHARLS) from 2011 to 2018 were included in this study. Physical activity levels were expressed as metabolic equivalents, and the impairment of daily physical function was determined in a self-reported format by the Activities of Daily Living Scale and the Instrumental Activities of Daily Living Scale. The association between different levels of physical activity and impaired daily physical function was analyzed using Cox proportional hazards regression models. Results: During a mean follow-up period of 6.73 years, 1379 middle-aged and older adults had impaired physical function. After adjusting for all covariates, participants with a physical activity volume (PAV) ≥ 600 MET-minutes/week had a 61% lower risk of impaired daily physical function than those who were physically inactive (HR = 0.39, 95% CI 0.35–0.44). Participants with a PAV of 1800–2999 MET-minutes/week had the lowest risk of impaired daily physical function (HR = 0.33, 95% CI 0.26–0.42). Subgroup analysis showed that participants with a PAV ≥ 600 MET-minutes/week had a greater reduction in the risk of impaired daily physical function among participants who were male, older than or equal to 65 years, and without respiratory disease compared to participants who were physically inactive. Conclusions: This study showed that a PAV ≥ 600 MET-minutes/week could reduce the risk of impaired daily physical function in Chinese middle-aged and elderly people. However, a higher PAV is not better; a PAV in the range of 1800–2999 MET-minutes/week can be more effective in preventing daily physical function impairment in Chinese middle-aged and elderly people.

## 1. Introduction

Physical function has been defined as the integration and translation of physiological stimuli into muscular actions [1], such as walking, maintaining balance, and staying standing. Physical function in middle-aged and older adults is an important determinant in maintaining and improving their functional capacity [2]. However, physical functions such as muscle strength, postural balance, and aerobic capacity continue to decline in middle-aged and older adults as they age [3,4]. Many studies have shown that impaired daily physical function has become a common health problem among middle-aged and elderly people [5]. In addition, impaired physical function was strongly associated with individual disability [6] and increased risk of mortality [7,8] and can also affect the ability of middle-aged and older adults to care for themselves and live independently [9], as they often require more daily assistance and health care [10,11]. Related research data showed that by 2020, the elderly population with impaired physical function in China had reached 43.75 million, and it is expected to rise to more than 90 million by 2050 [12]. China has become the world’s largest population of older adults with impaired physical function [13]. The serious harm caused by impaired physical function has brought a huge economic and social burden to society and has become a major public health problem [5].

Previous studies have shown that physical activity is an effective intervention to prevent impaired physical function in middle-aged and older adults, that higher levels of physical activity (PA) help maintain physical function in community-dwelling older adults [14,15,16,17,18], and that PA is also effective in delaying the progression of physical functional limitations or disability [19,20]. Results from several large epidemiological studies also showed that PA had a beneficial effect on the assessment of measures of physical functional performance such as balance [21], strength [22], and mobility [23]. Performing 5 min of moderate or vigorous physical activity was strongly associated with a 2% increase in physical function levels [15]. Related studies have shown that middle-aged and elderly people, who usually suffer from multiple chronic diseases, can benefit from PA on a regular basis even with physical functional limitations [24].

However, despite the widespread interest in the effectiveness of physical activity in avoiding or delaying impaired physical function, related studies have mostly been cross-sectional in design [15,25], which may limit the causal judgments regarding the relationship between PA and impaired physical function. Most previous studies have focused on moderate- or high-intensity physical activity exercise levels and have not taken into account low-intensity physical activity [26]. Some studies have shown that low-intensity physical activity also plays an important role in slowing down impaired physical function in middle-aged and elderly people [19], and most of the physical activities in middle-aged and elderly people are low-intensity physical activities [27]; thus, the role of low-intensity physical activity in slowing down impaired physical function in middle-aged and elderly people should not be ignored. Previous studies have mostly examined PA with a single variable and also lacked the combination of PA and multiple variables to explore. Furthermore, and more importantly, although PA may have beneficial effects on physical function, there may be differences between different PAVs in how much they reduce the risk of impaired physical function in middle-aged and older adults [28], and the optimal PA intervals required to infer these effects remain to be elucidated, especially in a large sample of Chinese middle-aged and older adults. Therefore, the purpose of this study was to explore the association between different physical activity volumes and impaired physical function in Chinese middle-aged and older adults, to assess the optimal PAV intervals that would allow them to avoid impaired physical function, and to provide evidence to support the reduction in the risk of impaired physical function in Chinese middle-aged and older adults. Accordingly, the following hypotheses are proposed in this study. Hypothesis 1: A higher PAV including low-intensity physical activity can significantly reduce the rate of impaired physical function in middle-aged and older adults. Hypothesis 2: The rate of impaired physical function varies among middle-aged and older adults with different PAVs, and the existence of an optimal PAV interval could more effectively reduce the risk of impaired physical function among middle-aged and older adults in China.

## 2. Methods

### 2.1. Study Design and Participants

Data for this study were obtained from the China Health and Retirement Longitudinal Study (CHARLS), a household interview survey conducted by the National Development Institute of Peking University to investigate the social, economic, and health status of community residents aged 45 years or older in China [29]. The CHARLS national baseline survey began in 2011 and used a multi-stage stratified whole-group sampling method to cover more than 17,000 people in 150 county-level units in 28 provinces and cities in China, with three follow-up surveys in 2013, 2015, and 2018 to collect information including sociodemographic, physical, and biological assessments and health-related information. The CHARLS was approved by the Biomedical Ethics Review Committee of Peking University (IRB00001052-11015), and all study subjects signed an informed consent form.

**Figure 1:**
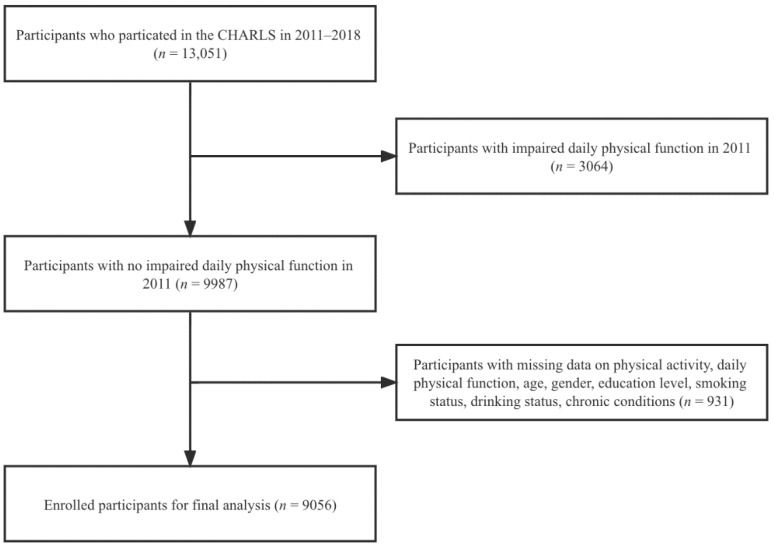
Flowchart of study participants.

This study combined health status and function data based on ID for a total of 4 years in 2011, 2013, 2015, and 2018. After merging, a total of 13,051 participants were selected in this study. Of these, a total of 9056 participants were ultimately included in the study analysis after those with impaired daily physical function in the 2011 baseline survey (*n* = 3064) and those with missing data for physical activity, daily physical function, and covariates (*n* = 931) were excluded (Figure 1).

### 2.2. Assessments

#### 2.2.1. Daily Physical Function

Daily physical function (DPF) was measured by the Activities of Daily Living (ADL) and the Instrumental Activities of Daily Living (IADL) scales [30]. The ADL scale covers dressing, bathing, eating, getting into/out of bed, using the toilet, and controlling bowel movements or urination. The IADL covers money management, taking medication, shopping, meal preparation, and household chores. Each question on both scales was divided into 4 levels, where “no difficulty” = 0, “difficulty but still able to complete” = 1, “difficulty and need help” = 2, and “unable to complete” = 3. A combined ADL/IADL score ≥11 was defined as “impaired physical function” and <11 was defined as “no impaired physical function” [28]. In subsequent analyses, DPF was defined as a dichotomous variable, assigned a value of 1 if physical function was impaired and 0 otherwise. The follow-up duration was determined as the time from the participant’s participation in the CHARLS (2011) to impaired physical function or to the termination of the fourth follow-up (2018), including 2 years, 4 years, and 7 years. The Cronbach’s alpha coefficient for the questionnaire was 0.792.

#### 2.2.2. Physical Activity

The questionnaire structure and statements used to measure participants’ PA levels were based on the International Physical Activity Questionnaire (IPAQ). In each of the four surveys from 2011 to 2018, participants were asked whether they had performed at least 10 min of vigorous physical activity (VPA, such as carrying heavy loads, digging, plowing, aerobic exercise, fast cycling, bicycling with cargo, etc.), moderate physical activity (MPA, such as carrying light things, cycling at regular speed, mopping, tai chi, brisk walking, etc.), and low-intensity physical activity (LPA, such as walking at work or at home and walking for recreation, exercise, or leisure). If available, further questions were asked about weekly frequency (1–7 days) and time spent per day for different PA levels (≥10 min and <30 min, ≥30 min and <2 h, ≥2 h and <4 h, and ≥4 h).

Since no specific duration was mentioned in the questionnaire, drawing on the treatment of other scholars [28], we transformed the time range by taking the middle value. That is, “≥10 min and <30 min” was recorded as 20 min, “≥30 min and <2 h” was recorded as 75 min, “≥2 h and <4 h” was recorded as 180 min, and “≥4 h” was recorded as 240 min.

The weekly duration scores for different PAs are the product of the weekly frequency of different PA levels and the time spent per day. The total physical activity volume (PAV) score can be expressed using the metabolic equivalent (MET) [31], which is calculated as follows: PAV = 8.0 × weekly vigorous physical activity duration score + 4.0 × weekly moderate physical activity duration score + 3.3 × weekly low intensity physical activity duration score. According to the IPAQ, physical inactivity (PI) was indicated if the total PAV did not reach 600 MET-minutes/week [32]. Therefore, the PAV in MET-minutes/week was divided into two major categories (0–599 and ≥600) and seven subcategories (0–599, 600–1199, 1200–1799, 1800–2999, 3000–5999, 6000–8999, and ≥9000).

#### 2.2.3. Covariates

The covariates in this study included (1) demographic variables, such as age, gender (male or female), and education level (high school and below and college and above); (2) lifestyle behavior variables, including smoking status (current smoking, former smoking, and never smoking), and drinking status (current drinking, former drinking, and never drinking); (3) chronic disease status variables, including endocrine system diseases (no or yes), brain/nervous system diseases (no or yes), cardiovascular disease (no or yes), respiratory diseases (no or yes), orthopedic diseases (no or yes), and other diseases (no or yes).

### 2.3. Statistical Analysis

The baseline characteristics of the participants are expressed by frequency (*n*) and percentage (%). Cox proportional hazards regression models were used to calculate hazard ratios (HRs) and 95% confidence intervals (95% CIs) to determine the association between different PAV levels and impaired physical function. To assess the potential confounding effects of different covariates on the association between PAV and impaired physical function, three models were developed, and the three sets of covariates were added sequentially to the three models. The adjusted variables in Model 1 were gender and age; the adjusted variables in Model 2 were based on Model 1 with the addition of education level, smoking status, and drinking status; and the adjusted variables in Model 3 covered all covariates. The interaction between PAV and potential covariates was tested in Model 3, and subgroup analyses were conducted by gender, age, education level, smoking status, drinking status, and chronic disease status. Finally, a sensitivity analysis was performed to test the robustness of the results: the Markov chain Monte Carlo imputation method was used, assuming that the variables in the interpolated model showed a multivariate normal distribution with joint effects, specifically using predicted mean matching, binary logistic regression, and multiple logistic regression filling. The sensitivity analysis was performed on 9987 participants after five interpolations for variables with missing values.

Stata 17.0 (Stata Corporation, College Station, TX, USA) was used for all statistical analyses.

## 3. Results

### 3.1. Demographic Characteristics

Of the 9056 eligible participants included in this study, 4491 (49.59%) were men and 4565 (50.41%) were women, and the majority were younger than 65 years old (57.37%); had a high school education or less (96.41%); never smoked (56.71%); never drank alcohol (61.75%); and did not suffer from endocrine system diseases (95.20%), brain/nervous system diseases (94.18%), cardiovascular disease (78.68%), respiratory diseases (94.14%), orthopedic diseases (93.93%), and other diseases (87.58%). The details are shown in Table 1.

### 3.2. Physical Activity and Physical Function Impairment Rate

**Figure 2:**
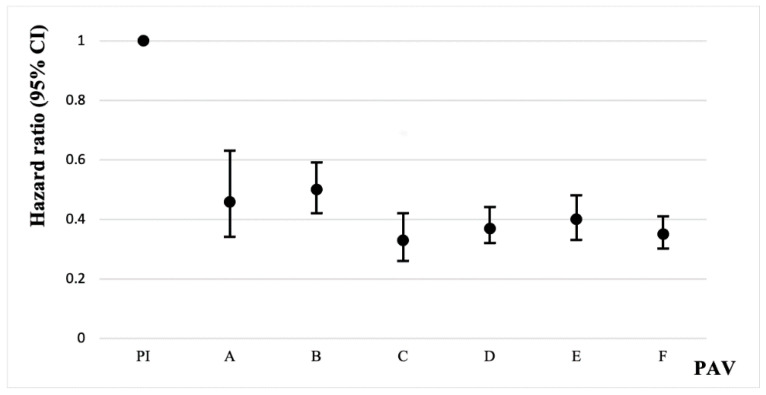
Plot of associations between study participants in different PAV groups and impaired physical function. Whiskers represent 95% confidence intervals. A: 600 ≤ PAV ≤ 1199. B: 1200 ≤ PAV ≤ 1799. C: 1800 ≤ PAV ≤ 2999. D: 3000 ≤ PAV ≤ 5999. E: 6000 ≤ PAV ≤ 8999. F: PAV ≥ 9000. Unit: MET-minutes/week.

A total of 1379 (15.23%) participants developed impaired physical function over the four follow-ups in the 7-year period (mean follow-up: 6.73 years) in 2011, 2013, 2015, and 2018. Table 2 and Table 3 show the correlation between different metabolic equivalents of PAV and impaired physical function. In both Model 1 and Model 2, participants with a PAV ≥ 600 MET-minutes/week had a 62% lower risk of impaired physical function compared to physically inactive participants (Model 1: HR = 0.38, 95% CI 0.33–0.42; Model 2: HR = 0.38, 95% CI 0.34–0.43). In the fully adjusted model (Model 3), participants with a PAV ≥ 600 MET-minutes/week had a 61% lower risk of impaired physical function compared to participants with insufficient physical activity (HR = 0.39, 95% CI 0.35–0.44). Among them, participants with a physical activity volume of 1800–2999 MET-minutes/week had the lowest rate of impaired physical function (Table 3 and Figure 2), and their risk of impaired physical function was 67% lower than that of physically inactive participants (HR = 0.33, 95% CI 0.26–0.42).

### 3.3. Subgroup Analysis

**Figure 3:**
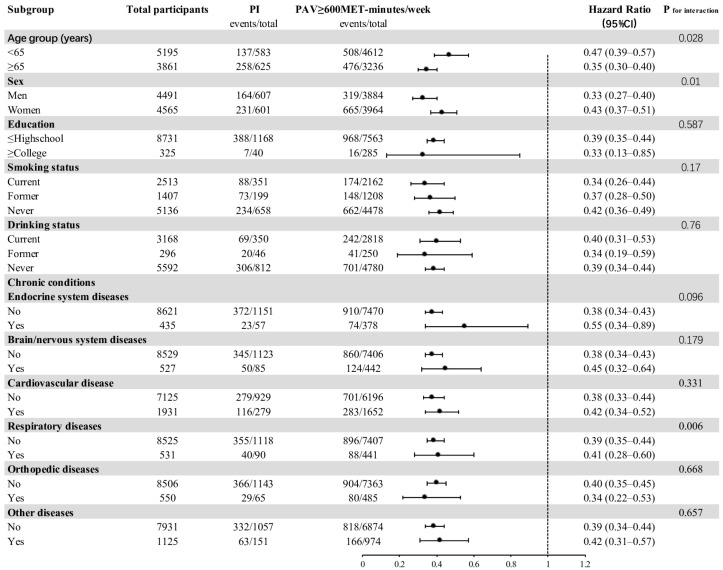
Subgroup analysis of the association between study participants with a PAV ≥ 600 MET-minutes/week and impaired physical function.

The association between physical activity level and impaired physical function was stratified by age, sex, education level, smoking status, drinking status, presence of endocrine system diseases, presence of brain/nervous system diseases, presence of cardiovascular disease, presence of respiratory diseases, and presence of other diseases, and the results of the subgroup analysis are shown in Figure 3. In the fully adjusted model, there was a significant interaction effect of age and physical activity level on impaired physical function (*p* = 0.028). Participants with a physical activity volume greater than 600 MET-minutes/week and aged 65 years or older had a lower risk of physical function deficits (HR = 0.35) than participants younger than 65 (HR = 0.47). There was a significant interaction effect of gender and physical activity level on impaired physical function (*p* = 0.01). The risk of physical function deficits was lower in male participants with a physical activity volume greater than 600 MET-minutes/week (HR = 0.33) than in female participants (HR = 0.43). In addition, there was a significant interaction effect of respiratory disease status and physical activity level on impaired physical function (*p* = 0.06).

### 3.4. Sensitivity Analysis

A sensitivity analysis was performed on the data to confirm the findings. The sensitivity analysis showed that excluding missing values for age, sex, education level, smoking status, drinking status, chronic conditions, and physical activity level at baseline had little effect on the risk estimate for impaired physical function (HR = 0.39, 95% CI 0.35–0.43).

## 4. Discussion

In this study, we found that a PAV ≥ 600 MET-minutes/week significantly reduced the risk of impaired physical function in Chinese middle-aged and elderly people. Moreover, a PAV in the range of 1800–2999 MET-minutes/week could more effectively reduce the risk of impaired physical function in Chinese middle-aged and elderly people.

This study found that higher levels of physical activity compared to physical inactivity helped to avoid or delay impairment of physical function in middle-aged and older adults. Hillsdon et al. [33] found that the decline in physical function with age was associated with low physical activity. Previous studies have also shown that aerobic exercise helps to improve cardiovascular health [34] while resistance exercise helps improve muscle strength, explosive power, flexibility, and balance [35,36], and that these improvements are essential to avoid or delay impaired physical function in middle-aged and older adults. Some studies have shown that the risk reduction from the effects of regular physical activity on various outcomes related to function is typically in the range of 30% to 50% [20]. This is generally consistent with the findings of this study that adequate physical activity can significantly reduce the risk of impaired physical function in middle-aged and older adults. Although loss of physical function is inevitable with age, early initiation of regular physical activity can reduce the age at which disability develops [37].

In order to help middle-aged and older adults engage in reasonable physical activity and promote health in this population, the World Health Organization (WHO) recommends that middle-aged and older adults should engage in at least 150 min of moderate-intensity PA or 75 min of high-intensity PA, or an equivalent combination of moderate-intensity and high-intensity aerobic PA each week [38,39]. However, most middle-aged and older adults may not meet the current recommendations [27,40]. Physical activity in middle-aged and older adults may be dominated by low-intensity physical activity, such as walking, compared to moderate- or high-intensity physical activity. It is true that meeting the WHO recommendations for physical activity plays an important role in avoiding impairment of physical function in middle-aged and older adults. However, when middle-aged and older adults are unable to meet this recommendation, our findings suggested that a PAV ≥ 600 MET-minutes/week, including low-intensity physical activity, can also be protective of physical function in this population. Hypothesis 1 was thus confirmed. This is consistent with previous studies showing that low-intensity physical activity can also slow the progression of physical disability in middle-aged and older adults [19,26].

It is well known that a higher PAV is not better. Previous studies have shown that excessive or high-intensity exercise impairs the immune system [41], which may adversely affect physical function in middle-aged and older adults. Several studies have also shown that overactivity is associated with reduced bone mineral density [42] and loss of muscle tissue [43]. Therefore, determining the optimal metabolic equivalent range of physical activity is essential to maintain or improve physical function. Studies have shown that for middle-aged and older adults with hypertension, PA of at least 1200 MET-minutes per week was associated with better physical function [28]. A metabolic equivalent of 1800–2999 MET-minutes per week significantly improved individual cardiopulmonary endurance, vascular endothelial function, and insulin sensitivity [44]. This is generally consistent with the findings of this study that 1800–2999 MET-minutes/week may be the optimal PAV to avoid impaired physical function in middle-aged and elderly Chinese people. Hypothesis 2 was thus confirmed. Although this study found that a PAV ≥ 9000 MET-minutes/week is also important for avoiding impaired physical function in middle-aged and elderly people, the possible adverse effects of excessive physical activity and the feasibility of implementing the recommended PAV in real-life situations should also be considered.

In terms of subgroup analysis, this study found that the negative association between PAV and the risk of impaired physical function in middle-aged and elderly Chinese was consistent across all subgroups. Additionally, it seemed to be more pronounced in participants who were 65 years old or older, male, and did not suffer from respiratory diseases. The reasons for this may be as follows: physical function declines gradually with age, and the effect of the decline becomes more pronounced with age, whereas physical activity counteracts the decline in physical function with age as much as possible [45]. It has been shown that middle-aged and older men are more likely to experience high-velocity muscle fatigue compared to middle-aged and older women [46], which may explain the higher reduction in the risk of impaired physical function associated with PA in middle-aged and older men than in middle-aged and older women. In addition, among middle-aged and older adults with a PAV ≥ 600 MET-minutes/week, those without respiratory diseases had a lower risk of impaired physical function compared to those with respiratory diseases, perhaps because the beneficial effects of physical activity were partially offset by respiratory diseases [47].

The present study has the following strengths: first, to our knowledge, it is the first study to examine the association between different physical activity levels and impaired physical function in middle-aged and older adults while considering multiple confounding factors and to explore the optimal PAV interval to avoid or mitigate impaired physical function in middle-aged and older adults in China. Second, this study was a longitudinal cohort study with a mean follow-up of 6.73 years for its sample. Third, this study used a large, nationally representative sample covering 28 provinces in mainland China, so the results of this study can be generalized to the entire Chinese middle-aged and elderly population. Furthermore, this database resource has been adopted by a large number of high-quality research institutes [48,49,50], with high validity and reliability. Fourth, considering the influence of confounding factors, this study added them to the model in turn, including demographic variables, life behavior variables, and chronic disease status variables. However, this study also has some limitations. First, PA was measured in a self-reported format, and self-report questionnaires are inevitably subject to recall bias. It has been shown that self-reported physical activity is on average overestimated compared to objective physical activity values measured by accelerometers [51]. For the measurement of PA, more objective measures such as the use of accelerometers are needed in the future. Second, survival time was derived from the difference between follow-up times. Since physical function was measured at the time of the questionnaire, the study could not determine the exact time of physical function impairment for individuals with impaired physical function. Therefore, only a rough estimate can be made due to the difference between the follow-up times.

## 5. Conclusions

Although current physical activity guidelines focus on moderate and vigorous physical activity, this study found that fulfilling a PAV ≥ 600 MET-minutes/week, including low-intensity physical activity (LPA, such as walking), also significantly reduced the risk of impaired physical function in Chinese middle-aged and older adults. In addition, a PAV in the range of 1800–2999 MET-minutes/week could be more effective in reducing the risk of impaired physical function in middle-aged and elderly Chinese.

## Figures and Tables

**Table 1:** Basic characteristics of participants.

Characteristics	Overall Sample (*n* = 9056)	
	*n*	%
**Age group (years)**		
<65	5195	57.37
≥65	3861	42.63
**Sex**		
Men	4491	49.59
Women	4565	50.41
**Education**		
≤High school	8731	96.41
≥College	325	3.59
**Smoking status**		
Current	2513	27.75
Former	1407	15.54
Never	5136	56.71
**Drinking status**		
Current	3168	34.98
Former	296	3.27
Never	5592	61.75
**Chronic conditions**		
**Endocrine system diseases**		
No	8621	95.20
Yes	435	4.80
**Brain/nervous system diseases**		
No	8529	94.18
Yes	527	5.82
**Cardiovascular disease**		
No	7125	78.68
Yes	1931	21.32
**Respiratory diseases**		
No	8525	94.14
Yes	531	5.86
**Orthopedic diseases**		
No	8506	93.93
Yes	550	6.07
**Other diseases**		
No	7931	87.58
Yes	1125	12.42

**Table 2:** Associations between study participants with a PAV ≥ 600 MET-minutes/week and impaired physical function. Values are hazard ratios (95% confidence intervals) unless otherwise noted.

Variables	Events/Total	Model 1	Model 2	Model 3
PI	395/1208	1.00	1.00	1.00
≥600	984/7848	0.38 (0.33–0.42)	0.38 (0.34–0.43)	0.39 (0.35–0.44)

Note: Events indicates the number of participants who showed impaired physical function. Model 1: Adjusted for sex and age. Model 2: Model 1 + education level, smoking status, and drinking status. Model 3: Model 2 + chronic conditions. PI: Physical inactivity.

**Table 3:** Associations between study participants in different PAV subgroups and impaired physical function. Values are hazard ratios (95% confidence intervals) unless otherwise stated.

Variables	Events/Total	Model 1	Model 2	Model 3
PI	395/1208	1.00	1.00	1.00
600–1199	46/295	0.44 (0.33–0.60)	0.45 (0.33–0.61)	0.46 (0.34–0.63)
1200–1799	209/1283	0.48 (0.41–0.57)	0.49 (0.42–0.58)	0.50 (0.42–0.59)
1800–2999	77/676	0.33 (0.26–0.42)	0.33 (0.26–0.43)	0.33 (0.26–0.42)
3000–5999	240/1974	0.35 (0.30–0.41)	0.36 (0.31–0.43)	0.37 (0.32–0.44)
6000–8999	151/1125	0.39 (0.32–0.47)	0.40 (0.33–0.48)	0.40 (0.33–0.48)
PAV ≥ 9000	261/2495	0.33 (0.28–0.39)	0.34 (0.29–0.40)	0.35 (0.30–0.41)

Note: PI: Physical inactivity. PAV: Physical activity volume.

## Data Availability

Publicly available datasets were analyzed in this study. These data can be found at http://charls.pku.edu.cn/, accessed on 10 October 2022.
